# EDUCATION AND TRAINING ELECTRONIC NAVIGATION SYSTEMS’ IMPACT ON OLDER DRIVER PERFORMANCE AND TRAINING TO OVERCOME UNFAMILIARITY

**DOI:** 10.1093/geroni/igz038.2460

**Published:** 2019-11-08

**Authors:** Anne E Dickerson, F Dennis Thomas, Lindsay Graham, M Chandler Coleman, Richard Blomberg, Tim Wright

**Affiliations:** 1 East Carolina University, Greenville, North Carolina, United States; 2 Dunlap and Associates, Stamford, Connecticut, United States

## Abstract

Technology may assist older adults in improving their driving performance and therefore driving safety. However, it is sometimes a distraction and some older adults avoid its use due to the complexity of learning the systems. This study examined how older drivers interacted with an electronic navigation system (e.g., GPS) and the extent to which it impacted driving performance on unfamiliar routes. It also examined three approaches to training older adults how to program the devices. In Phase 1, 80 older drivers navigated unfamiliar routes using a GPS or paper directions and completed destination entry tasks. In Phase 2, 60 older drivers completed one of three training conditions (video, video with hands-on, placebo) to examine the impacts of training on destination entry performance. Driving performance was improved with GPS over paper directions (p = .025), as evaluated by a driver rehabilitation specialized on counterbalanced standardized routes. Analyses also showed significant effects for familiarity for use of GPS (p=.035) and age group (60’s versus 70’s) (p<.001), but many drivers had difficulty entering destinations. In Phase 2, a main effect of training was found (p=.02) with using video and one-on-one training showing the best performance. This study demonstrates older drivers could benefit from the use of such devices when driving to unfamiliar destinations, but training is needed with hands-on training with a live instructor being the best.

